# Emily Elizabeth Eberle

**Published:** 1908-12

**Authors:** 


### Obituary Notices.

### EMILY ELIZABETH EBERLE, M.A., M.B., B.CH., B.A.O., F.R.C.S.

DR. EMILY EBERLE died on September 12th at Dublin. She had undergone a severe abdominal operation fifteen days previously, and appeared to be recovering with unusual rapidity, when symptoms of tetanus set in on the thirteenth day and quickly proved fatal.

Emily Eberle was the daughter of a Moravian minister of Dublin, and was educated up to the age of fifteen years at Fulneck, Yorkshire, a school founded for the children of Moravians. On the death of her father in 1876 she returned to Dublin, and attended the Alexandra College for girls, and then passed on to the classes for women at Trinity College, obtaining in 1880 a certificate of honours in languages and botany, and in 1882 a like certificate and also the prize in mathematics. In 1884 she graduated as Bachelor of Arts at the Royal University of Ireland, and devoted her energies for some years to teaching Latin and mathematics in schools for girls. The death of her mother in 1887 called her to fill a vacant place at home with a brother and sister. Domestic duties, however, did not absorb all her time, for she obtained the degree of Master of Arts in 1889 at the Royal University of Ireland. Her attention was then directed by an eager friend to the medical profession, and her energies found new paths in working for a medical degree. In 1894 she graduated as Bachelor of Medicine, Obstetrics and Surgery, and was also Licentiate of Midwifery. The death of the brother with whom she lived severed her last tie to Dublin, and at the invitation of another brother she came to Bristol, where members of her family were widely known and esteemed.

Dr. Eberle threw herself with much energy into medical practice. She joined the Read Dispensary Staff, opened also a dispensary at Bedminster for a few months, and accepted service at the Bristol Private Hospital, which was opened in December, 1895. She applied for and obtained in 1897 the post of Assistant Surgeon at the Hospital for Children and Outdoor Treatment of Women, to which appointment was attached the condition of acquiring within a year the Fellowship of a College of Surgeons. This condition she successfully accomplished at the Royal University of Ireland. At the Children's Hospital she attracted a large number of patients, and also much of the gynæcological out-patient work was handed over to her.

As private practice increased it became necessary to curtail her public service, and she resigned her post at the Children's Hospital, retaining her visiting days at the Read Dispensary and her post at the Bristol Private Hospital.

Dr. Eberle brought to her medical practice quick intelligence, uncommon energy, a power of lively sympathy and good business ability. She concentrated all her powers upon her profession, and passed laborious days devoted to it alone, knowing little or nothing of pleasures outside those of the family circle. Her sympathy and interest in the sick never flagged, and ailing women of all classes and from a wide area availed themselves in a large number of her medical help, given with ungrudging expenditure of time, thought and warm sympathy.

Her colleagues of the Read Dispensary and Private Hospital for Women lament the loss of the single-eyed devotion and alert intelligence which she brought to her work, and know that their loss will not be easily repaired.

